# Supplementary material for: Revisiting the origins of the Sobemovirus genus: A case for ancient origins of plant viruses
Source: PLoS Pathog. 2024 Jan 11;20(1):e1011911. doi: 10.1371/journal.ppat.1011911 (PMC10807823; doi:10.1371/journal.ppat.1011911)
Supplement: S1 Table — (DOCX) [file ppat.1011911.s001.docx]

| **Accession number** | **Sample collection date** |
| --- | --- |
| AB040447 | 1978 |
| MAFF307042 | 1978 |
| MAFF104073 | 1981 |
| AB047688 | 1982 |
| MAFF104072 | 1982 |
| DQ680848 | 1985 |
| NC002618 | 1985 |
| MAFF307050 | 1988 |
| MAFF307051 | 1988 |
| MAFF307049 | 1989 |
| FJ669143 | 1990 |
| AJ243469 | 1995 |
| AJ243470 | 1995 |
| AJ243471 | 1995 |
| AJ243472 | 1995 |
| AJ243473 | 1995 |
| L40905 | 1995 |
| GU002336 | 2001 |
| EF422382 | 2005 |
| EF422383 | 2005 |
| EF422384 | 2005 |
| EF422385 | 2005 |
| EF422386 | 2005 |
| EF422387 | 2005 |
| EF422388 | 2005 |
| EF422389 | 2005 |
| EF422390 | 2005 |
| EF422391 | 2005 |
| EF422392 | 2005 |
| EF422393 | 2005 |
| EF422394 | 2005 |
| EF422395 | 2005 |
| EF422396 | 2005 |
| OR509655 | 2008 |
| MW147174 | 2014 |
| KT984653 | 2015 |
| KT984654 | 2015 |
| KT984655 | 2015 |
| KT984656 | 2015 |
| KT984657 | 2015 |
| KT984658 | 2015 |
| KT984659 | 2015 |
| KT98466 | 2015 |
| KX880413 | 2016 |
| MF621330 | 2016 |
| MW588040 | 2016 |
| MG765457 | 2017 |
| MG765458 | 2017 |
| MG765459 | 2017 |
| MG765460 | 2017 |
| OQ974933 | 2022 |
