# Supplementary material for: Revisiting the origins of the Sobemovirus genus: A case for ancient origins of plant viruses
Source: PLoS Pathog. 2024 Jan 11;20(1):e1011911. doi: 10.1371/journal.ppat.1011911 (PMC10807823; doi:10.1371/journal.ppat.1011911)
Supplement: S2 Table — (DOCX) [file ppat.1011911.s002.docx]

| **Virus name** | **Number of isolates** | **Min. genetic identity (%)** | **Accession numbers of the most divergent pair of isolates** | |
| --- | --- | --- | --- | --- |
| Cocksfoot mottle virus | 7 | 95.0 | L40905 | MW197136 |
| Cymbidium chlorotic mosaic virus | 6 | 90.6 | KR996515 | ON286969 |
| Lucerne transient streak virus | 4 | 98.0 | NC001696 | OK181163 |
| Physalis rugose mosaic virus | 2 | 97.4 | MK681145 | MK681145 |
| Pistacia sobemovirus | 4 | 93.0 | MT334603 | MW548580 |
| Rice yellow mottle virus | 72 | 92.1 | MF989228 | MZ172959 |
| Ryegrass mottle virus | 7 | 98.6 | MT129760 | MW411579 |
| Southern bean mosaic virus | 20 | 90.8 | AF055888 | OK670700 |
| Southern cowpea mosaic virus | 2 | 91.8 | MZ355623 | NC001625 |
| Sowbane mosaic virus | 6 | 95.0 | GQ845002 | OK558770 |
| Subterranean clover mottle virus | 10 | 98.6 | OM818392 | OM818396 |
| Turnip rosette virus | 4 | 91.9 | KC778720 | KC778721 |
