# Supplementary material for: Revisiting the origins of the Sobemovirus genus: A case for ancient origins of plant viruses
Source: PLoS Pathog. 2024 Jan 11;20(1):e1011911. doi: 10.1371/journal.ppat.1011911 (PMC10807823; doi:10.1371/journal.ppat.1011911)
Supplement: Attachment — Submitted filename: response to reviewers.docx [file ppat.1011911.s003.docx]

We thank the reviewers for their careful read of our manuscript and their helpful and constructive comments. We have addressed all their concerns and as a result we believe that the manuscript has considerably improved.

Please find below our response to the reviewers’ comments.

**Reviewer #1:**

This very interesting study revises the evolutionary history of sobemoviruses by taking into account the TDRP. As a result it is proposed that the origin of this genus, and its initial diversification on infect different plant families, is orders of magnitude older than previously considered. The study is based in all available information on sobemovirus diversity, and uses the most appropriate methodology, and the paper is well written and easy to read. I have only minor suggestion that could improve the text.

We thank the reviewer for their insightful and positive assessment of our study. We are glad to hear that our efforts to revise the evolutionary history of sobemoviruses, incorporating the TDRP, and our methodology have been well-received.

1. Abstract and elsewhere. The authors often refer to the Neolithic as a time frame, which may be confusing and is probably incorrect. The neolithic refers to a period of technical development associated with agriculture, and occurs at different periods in different regions of the world, the 10000 yr PB mentioned is mostly of application to the Near East. As sobemoviruses seem to have “speciated” in different regions of the world (as stated for CfMV and RYMV) the time frame for the neolithic may vary very much. Certainly, neither in West Africa nor in temperate Europe did the neolithic occur 10000 yr ago. It would be thus good to rephrase the corresponding sentences.

Thank you for pointing out the imprecision in our use of the term 'Neolithic.' We acknowledge the variability in the timing of the Neolithic period across different regions, which is especially relevant given the global diversification of sobemoviruses we discuss. We have now revised the relevant sections of our manuscript to ensure that our references to time frames are accurate and clear. We further clarified, in the introduction, that the Neolethic occurred at different periods in different regions, with appropriate references added.

New references:

Ammerman AJ, Cavalli-Sforza LL. Measuring the rate of spread of early farming in Europe. Man (Lond). 1971;6: 674.

Gangal K, Sarson GR, Shukurov A. The near-eastern roots of the Neolithic in South Asia. PLoS One. 2014;9: e95714.

2. Lines 111.113. No need to use three independent sentences all starting by “The genome”.

We agree. We have revised these sentences to eliminate the repetition and improve the flow of the text.

3. Metagenomic sequences. I am surprised that no sequences were retrieved from plant viromes or plant metagenomic data. How is this so? Were plant data bases not searched?, or they were searched and no sobemovirus sequences were found?

Indeed, our metagenomic sequences includes several species that have been discovered or sequenced using high-throughput sequencing of plant viromes. We apologize if this was not made clear. We have now amended Table 1 to explicitly highlight those sequences.

4. Line 188. Herbaceous should be substituted by herbivore.

Now revised.

5. Discussion. The uncertainties unavoidably associated to phylogenetic analyses extended back in time, and shown by the huge confidence intervals in Fig. 3a, should be somehow acknowledge in the discussion. Such uncertainties, by the way, do not in the least diminish the interest and novelty of the results, as they are intrinsic to all historic studies.

We thank the reviewer for highlighting this important point. We have now added a new paragraph in the discussion which acknowledges the inherent uncertainty of our model predictions, highlighting the challenges and limitations of reconstructing deep evolutionary histories.

6. The colours used to indicate host families in Fig. 2b are really hard to see. Perhaps the colours could be used as shadows over the virus name rather than in the letters.

We have updated the tree in Figure 2b as per the reviewer's request, so now the host names are indicated in light shaded colors over the virus names.

**Reviewer #2:**

In this interesting and potentially important paper, Ghafari et al. estimate the age of the viruses in the family Sobemoviridae, taking into account the time-dependent rate phenomenon using the Prisoner of War model.

The come up with an estimate of about 4 million years for the origin of sobemoviruses, orders of magnitude older than previously believed. They then explore the evolutionary history of this virus family, revealing likely host jumps and major radiation during the Neoltithic period. Such findings are indisputably important for understanding virus evolution. The article is very clearly and well written but, in the opinion of this reviewer, requires a substnatial amendment to become fully convincing, as detailed below.

We are grateful to the reviewer for their positive and insightful assessment of our work. In response to the points raised, we have made substantial amendments to the manuscript. These revisions include a more detailed clarification of the Prisoner of War (PoW) model in both the introduction and, more extensively, in the methods section. Additionally, we have expanded the discussion to provide greater context on the broader implications of our findings for understanding the deep evolutionary history of viruses. We believe these revisions address the main concerns raised and also enhance the clarity of our methodology and the overall readability of the paper.

Surprisingly, nowhere in the paper is the Prisoner of War model actually explained. This seems to be a must for the reader to be able to understand what was actually done. An adequate explanation has to be included in the Introduction. Even more importantly, the description of the model in Methods section is overly brief and effectively uninformative. It needs to be adequately expanded including the statistical evaluation of the results. As it stands, the reader simply has to believe the estimates which is not acceptable.

We appreciate the reviewer's crucial point regarding the lack of explanation for the PoW model in our paper. We apologize for this oversight and have taken steps to rectify it in the revised manuscript. More specifically, we have dedicated a paragraph in the introduction to outlining the fundamental principles of the PoW model and its application to estimating evolutionary history of viruses. We have also completely re-written the section of the methods which explains the PoW model to include detailed explanations of the model parameters and the statistical evaluation of our results. These additions are aimed at providing readers with a clear understanding of our methodology, ensuring that all necessary information to understand the model is accessible within the paper.

I suppose this is too much to ask but to this reviewer, this work would have been far more interesting and compelling if more than one virus family was analyzed and compared. Furthermore, can this analysis be extended to estimate the timeline of virus evolution deeper, beyond the family level? Hopefully, at least, this will be addressed in future work. Perhaps, worth mentioning in the Discussion.

We appreciate the reviewer's suggestion and recognize the value in expanding our analysis to include more than one virus family. The application of the PoW model to a broader range of virus families indeed presents major challenges, particularly in data collection and the establishment of consistent assumptions across different viruses. Extending this analysis beyond the virus family level, for instance, introduces additional complexities, such as aligning highly divergent sequences. However, these are important areas of ongoing research for us, and while we believe they fall outside the scope of the current work, they are certainly on our radar for future studies.

More specifically, one such area for future work would be to date the diversification at both the family (Solemoviridae) and interfamily level (Solemoviridae and Barnaviridae), as the reviewer rightly suggested. We think some of the alignment challenges can be overcome by considering the structural properties of the polymerase to be able to reliably construct phylogenetic trees and infer divergence times. Another area of potential interest would be to investigate the history of the *Polerovirus* genus within the Solemoviridae family which will parallel our study with *Sobemovirus* genus, but will focus on a genus with different ecology, more species, and greater economic importance. On a more general point, we hope this work will encourage the experts in the field to make a collective effort in reassessing key phylogeographic studies of plant viruses that may have erroneously extrapolated short-term substitution rates to longer periods and, therefore, underestimated the evolutionary history of some plant viruses.

We have now added a few paragraphs in the discussion, explaining these points in more detail.
